# Placing Workplace Wellness in Proper Context: Value Beyond Money

**DOI:** 10.5888/pcd11.140128

**Published:** 2014-07-10

**Authors:** Nicolaas P. Pronk

Most companies in the United States now offer some kind of wellness programming to their employees. In 2012, about half of US employers with at least 50 employees and more than 90% with more than 50,000 employees offered a workplace wellness program (1).

Employer surveys (eg, the 2011 Automatic Data Processing Survey) suggest that the most often-cited reasons for offering these programs include improved employee health, health care cost control, increased productivity, and absenteeism reduction. Each of these reasons is quantifiable, and their value can be monetized, allowing for a calculation of savings and an estimation of a return on investment (ROI).

Yet it isn’t necessarily always about saving money. Dr Risa Lavizzo-Mourey, president and chief executive officer of the Robert Wood Johnson Foundation, notes in a recent online post that companies committed to nurturing a culture of well-being consider broader motivations, including low turnover rates, attraction of top candidates, job satisfaction, and recruitment and retention of workers (2). Each factor was reported as being a more important driver of workplace wellness programs than ROI.

There is understandable interest in learning if, and how, workplace wellness programs produce results and generate savings. Publications on the effect of workplace wellness on financial outcomes continue to accumulate (1–5), but instead of producing consistency and clarity, they have introduced doubt and controversy. Systematic reviews and meta-analytic findings indicate that workplace wellness can generate savings (3). However, recent ROI studies indicate that such savings come only from disease management (DM) programs not lifestyle management (LM) programs (1). To gain clarity, 2 issues must be addressed. First, there is a need to standardize the definition of workplace wellness programs so that casual use of what constitutes such programs can be avoided. Second, research approaches should more explicitly recognize that workplace wellness programs generate a range of outcomes, many of them non–health related, that provide substantial value to employers even though they are often not represented in ROI analyses. 

This essay addresses these 2 issues in the context of a set of best practice program-design principles that allow for properly designed workplace wellness programs to be differentiated from other activities that, although well-intended, may not rise to the level of a bona fide program.

## Workplace Wellness Defined

A recent survey noted that 77% of employers offering health benefits also offered a workplace wellness program (6). These programs were defined as providing access to at least 1 of the following services: weight loss, gym membership discount or on-site exercise facility, smoking cessation, health coaching, classes in nutrition or healthful living, biometric screening, Web-based resources for healthful living, a wellness newsletter, influenza (flu) shots or vaccinations, or an employee assistance program (EAP). According to this definition, flu shots as a single activity may be counted as a workplace wellness program. Alternatively, *Healthy People 2010* defined comprehensive worksite health promotion programs as programs that provide 1) health education, 2) supportive social and physical environments, 3) integration into the organization’s structure, 4) links to related programs like EAPs, and 5) worksite screenings (7). Only 6.9% of US companies met these criteria in 2004 (7). Whereas the *Healthy People 2010* definition is more comprehensive than merely considering any single health promotion activity, many identified best practice program-design elements are not included. Recent work categorized best practice elements into principles for successful workplace wellness programs and noted that the *Healthy People 2010* definition represents only 1 of 9 best practice dimensions (Box). Clearly, not all workplace wellness programs are created equal. It seems reasonable to be more explicit in describing how closely any program adheres to such design principles to place research results in proper context.

**Box. Best Practice Design Dimensions for Workplace Wellness Programs. Adapted from Pronk (8).**

**Leadership** — design elements that set program vision, set organizational policy, ensure resources, support implementation, and connect the program to business goals.

**Relevance** — design elements that address factors critical to participation and employee engagement.

**Partnership** — design elements that relate to efforts that integrate the program with other groups or entities, including employees, unions, external vendor companies, and community organizations, among others.

**Comprehensiveness** — design elements that address health education, supportive social and physical environments, integration of the worksite program into the organization’s structure, links to related programs, and worksite screening programs.

**Implementation** — design elements that ensure a planned, coordinated, and fully executed work plan and process tracking system.

**Engagement** — design elements that promote an ongoing connection between employees and the program through actions that create respect, trust, and an overall culture of health and well-being.

**Communications** — design elements that ensure a strategic communications plan that generates a day-to-day presence of the program in the workplace.

**Being Data-Driven** — design elements that ensure the use of data in measuring, integrating, evaluating, and reporting program evolution and continuous improvement efforts.

**Compliance** — design elements that ensure the program meets regulatory requirements and protects personal information of employees and participants.

Any definition should, at a minimum, recognize workplace wellness as a population health strategy — programs, policies, and systems applied to the entire workforce, and often their families — and an appreciation of relevant macro forces that influence and shape the lives of people. For example, the Centers for Disease Control and Prevention’s workplace health initiative provides the following definition:

Workplace health programs are a coordinated and comprehensive set of health promotion and protection strategies implemented at the worksite that includes programs, policies, benefits, environmental supports, and links to the surrounding community designed to encourage the health and safety of all employees (9).

This definition clearly identifies workplace wellness as a population-based strategy. However, it is less specific about the broader context that affects such programs, including the effect of macro forces such as the market dynamics of a company’s industry, labor market forces, the impact of social influences (eg, religion, health disparities, or political viewpoints) on its workforce, or changes in the health insurance landscape. Such macro forces have separate but related effects on the health outcomes of the population being considered.

## Value Beyond ROI

When best practice principles are applied to workplace wellness program design, successful outcomes are more likely to occur (4). The value workplace wellness programs bring also comes from outcomes beyond financial or economic factors, such as physical and mental health, quality of life, perceived health status, and functional capacity (4,7). Furthermore, companies may place value on other factors that are non–health related. On the basis of work published by the Institute of Medicine (10) and adapted to the workplace setting, factors may stem from “workplace well-being” or “workplace process” value components. Workplace well-being components may include examples such as social cohesion of work teams, access to health care benefits, and a physical activity–friendly work environment. Workplace process components include such factors as skill development, participatory approaches to employee engagement, worker representation on committees, and involvement in decision making. Monetizing the value of increased social cohesion of a work team or the pride, trust, and respect that comes from being actively engaged in the company’s health and wellness program may not be possible. Yet, these factors carry inherent value to organizations.

## Context Matters

When the definition of workplace wellness programs is applied to subgroups instead of an entire population, conflicting results may emerge. One example is a recent analysis of the workplace wellness program at PepsiCo (1). This study followed employees enrolled in a LM program, a DM program, or a combined (LM + DM) program for 7 years. The study presented very little information related to methodological considerations. For example, it did not provide information on the crossover between DM and LM programs or the turnover of employees during the study period. Furthermore, minimal information was provided on program design, so the degree to which best practice design dimensions were met cannot be discerned from the article.

The overall PepsiCo program improved health and generated a positive ROI of $1.46 for every dollar invested; however, subgroup analyses indicated that net savings could be attributed only to the DM component, not the LM component. However, this is a departure from considering workplace wellness as a population health strategy. Best practice DM programs integrate healthy lifestyle and behavior change components — well-known elements of LM programs. DM programs represent a hybrid of wellness and condition management services, which makes problematic the study’s conclusion that LM programs do not save money. This argument is substantiated in the study because the data indicate that the combined group actually generated higher savings than the DM-only group. When an LM program subgroup is defined as employees who have lower health risks and, consequently, lower health care costs, it should hardly be surprising that this group does not generate substantial savings. Hence, evaluation of DM and LM separately does not truly test the effectiveness of an overall workplace wellness program.

Various interpretations of study results communicated in the media and via opinions of others (5,11) cast doubt on the value of workplace wellness programs. In general, researchers and those who communicate study results by using lay media should use caution when interpreting results on the basis of segmentation of populations into components that fundamentally alter the definition of workplace wellness programs and, as a consequence, the promise of such programs to generate savings.

## The Real Value of Workplace Wellness

A healthy, well, resilient, and vital workforce may be considered a corporate asset in striving toward a healthy bottom line. It is also an important piece in the puzzle of creating healthy, vibrant, and productive communities. Those healthy communities stand at the heart of a vital local economy that attracts new employers and industries, creates jobs, increases housing values, enhances prosperity, and supports local, national, and global competitiveness. These observations highlight important connections between public agencies and private industry, regional economic development efforts and corporate leadership, and the monitoring of important health indicators through public health surveillance and corporate performance, just to name a few factors. Research designed to study the value of workplace wellness programs should be guided by comprehensive assessments of all benefits, harms, and resources used and placed in its proper population health context to derive clear conclusions. Doing so will prove helpful and constructive in shaping appropriate expectations for what these programs can deliver.
